# Outcomes of Bonebridge Implantation in 10 Patients with Rare Genetic Syndromes and Difficult Anatomy

**DOI:** 10.3390/jcm15083064

**Published:** 2026-04-17

**Authors:** Katarzyna B. Cywka, Piotr H. Skarzynski, Emilia A. Czaplicka, Henryk Skarzynski

**Affiliations:** 1 Otorhinolaryngosurgery Clinic, World Hearing Center, Institute of Physiology and Pathology of Hearing, 02-042 Warsaw, Poland; 2 Department of Teleaudiology and Screening, World Hearing Center, Institute of Physiology and Pathology of Hearing, 02-042 Warsaw, Poland; e.czaplicka@ifps.org.pl; 3 Institute of Sensory Organs, 05-830 Nadarzyn, Poland; 4 Ear Nose and Throat Department, Center of Hearing and Speech MEDINCUS, 05-830 Kajetany, Poland

**Keywords:** microtia, atresia, conductive hearing loss, mixed hearing loss, bone conduction implant, genetic syndrome

## Abstract

**Background**: Congenital hearing loss occurs in about 2 of every 1000 newborns, of which half probably have a genetic origin. In syndromic patients, hearing impairment often results from craniofacial malformations affecting the outer and middle ear. Anatomical limitations such as microtia or external auditory canal atresia often preclude conventional air-conduction hearing aids, leaving bone-conduction devices as one viable option. However, surgical intervention in such patients is challenging. This study aimed to evaluate the audiological outcomes, safety, and effectiveness of the Bonebridge BCI 602 implant in 10 patients with genetic syndromes. **Methods**: The case series was made up of 10 patients aged 6–45 years, each diagnosed with a congenital syndrome affecting the external and/or middle ear. All cases involved surgical implantation of the Bonebridge system. Audiological outcomes were evaluated in free-field conditions on the day of sound processor activation and at 3–6 months follow-up via pure-tone and speech audiometry. **Results**: All surgical procedures were completed without serious adverse events, and the incidence of postoperative complications was low. Audiological outcomes showed clinically significant hearing improvement in all patients following Bonebridge implantation. Post-implantation hearing thresholds ranged from 25 to 40 dB HL, with notable gains in speech perception in both quiet and noisy environments. **Conclusions**: The Bonebridge implant appears to be a safe and effective option for auditory rehabilitation in patients with hearing loss associated with various genetic syndromes involving craniofacial malformation. However, this complex patient population requires individual assessment, interdisciplinary evaluation, and careful surgical planning.

## 1. Introduction

Genetic syndromes are a group of disorders caused by abnormalities in an individual’s genome. The changes may be hereditary, meaning the abnormality is passed down from generation to generation, or they may result from a de novo mutation that arises before fertilisation in the reproductive cell of a parent [1,2]. The abnormalities can lead to disorders affecting various body structures and systems, including anatomical, physiological, and neurological defects. The clinical presentation of genetic syndromes varies depending on the type of mutation and the extent of affected systems and organs [3,4].

Genetic syndromes involving defects of the auditory system are particularly prominent, since about 1% of the human genome is involved in hearing, corresponding to an estimated 300–500 genes [5]. Congenital hearing loss occurs in about 1.86 of every 1000 newborns [6,7,8,9], and it is estimated that approximately 50% of cases of prelingual hearing loss have a genetic origin. Some 80% of patients with genetically determined prelingual hearing loss have no additional abnormalities involving other organs or systems, a condition referred to as isolated (nonsyndromic) hearing loss [10]. When additional anatomical or functional abnormalities are present, hearing loss is classified as syndromic [2,8,11]. Hearing impairment in patients with syndromic hearing loss most commonly results from craniofacial malformations and outer and/or middle ear abnormalities [8,12,13]. Syndromes most frequently associated with these defects include Treacher–Collins syndrome (TCS), CHARGE syndrome, Goldenhar syndrome (GS), and other mandibulofacial dysostoses (MFDM). All of these conditions are classified in the NORD (National Organization for Rare Disorders) database, which focuses on rare diseases [14]. These syndromes are rare, with most occurring in fewer than 1 in 10,000 live births [15,16,17]. Despite their differences in their genetic background, clinical presentation and associated comorbidities, these syndromes share common anatomical features involving the external and/or middle ear. These features include microtia, atresia of external auditory canal, and ossicular malformations, which predominately lead to conductive hearing loss. Hearing impairment is highly prevalent in this group, affecting 50% to 100% of patients, depending on the specific syndrome [2,3,16,18]. In some cases, inner ear involvement is also present, resulting in mixed hearing loss. In some cases, inner ear involvement may be present, resulting in a wide range of hearing loss types and severities [19,20,21]. However, conductive hearing loss is most common, while additional inner ear pathology results in mixed hearing loss [2,17,18].

Early and accurate diagnosis of genetic syndromes is essential because it enables multidisciplinary care and appropriate genetic counseling. An accurate diagnosis enables effective treatment strategies that can significantly improve the patient’s quality of life [12]. In cases of congenital hearing loss, early diagnosis is crucial. According to the 2019 recommendations of the Joint Committee on Infant Hearing, diagnosis should be done within 2 months of age. Intervention involving hearing aids or cochlear implants should be initiated by 3 months of age [22]. Due to anatomical limitations, such as microtia or atresia of the external auditory canal, conventional air-conduction hearing aids are often not feasible. Bone-conduction hearing devices (BCDs) can be an effective alternative in such cases. It is important to note that, in cases of mixed or sensorineural hearing loss, BCDs do not restore hearing sensitivity at frequencies where deficits are present [15,20,23].

One such device is the Bonebridge system (Med-El, Innsbruck, Austria). This device has been demonstrated to be effective in numerous clinical studies, also in patients with abnormal craniofacial anatomy [24,25,26]. The system consists of an external sound processor linked to an internal vibrator beneath the skin that is surgically implanted within the temporal bone. Its design means that sound transmission bypasses the outer and middle ear, allowing acoustic stimuli to reach the inner ear directly. The Bonebridge implant is approved for adults and children over 5 years old with conductive or mixed hearing loss [25,27]. In children under 5 years old, external bone-conduction systems are typically fitted. These systems include the Ponto and BAHA devices (mounted on a soft headband), and the AdHear adhesive system [26,28].

Studies indicate a low incidence (only 1–5%) of intraoperative complications, adverse events and need for revision associated with Bonebridge implantation [24,27]. However, surgical difficulties can arise in patients with atypical temporal bone anatomy, a condition frequently seen in individuals with genetic syndromes. In such cases, detailed preoperative planning of the internal component of the implant is essential, taking into account the thickness of the temporal bone and the available space [23]. Compared with the previous BCI 601 version, the BCI 602 facilitates implantation and provides improved stability within the temporal bone. The most important difference is reduced thickness of transducer (from 8.7 to 4.5 mm). In addition, flexible transition segment, allows better adaptation to patient’s individual and abnormal anatomy. Its smaller size and more ergonomic design allow it to be used in patients with anatomical abnormalities for whom implantation with the BCI 601 was not possible [29,30]. Because the implant needs to be placed close to critical structures such as the sigmoid sinus and dura mater, careful surgical planning is required based on computed tomography (CT) and/or magnetic resonance imaging (MRI), together with otologic planning software (e.g., Otoplan) [31,32]. Surgery on children with congenital craniofacial deformities poses significant operative challenges, so careful evaluation of the indications for Bonebridge implantation, as well as a thorough assessment of its potential clinical benefits, is required.

This study aims to evaluate the surgical considerations, audiological outcomes, safety, and effectiveness of the Bonebridge BCI 602 implant in patients with genetic syndromes associated with hearing loss and craniofacial malformations.

## 2. Materials and Methods

### 2.1. The Study Group

The case series comprised 10 patients from across Poland (6 women and 4 men) ranging in age from 6 to 45 years; 3 of them were adults and 7 were children. The patients had been diagnosed at genetic clinics with the following conditions: TCS (n = 5), GS (n = 2), Klippel-Feil syndrome (KFS) (n = 1), CHARGE syndrome (n = 1), and MFDM (Guion–Almeida type) (n = 1).

### 2.2. Study Design

At the preoperative evaluation for an implantable bone conduction device, patients underwent a comprehensive audiological assessment including pure-tone and speech audiometry. To assess their sensitivity to bone-conducted sound, bone conduction hearing aids on headbands (Ponto System Oticon Medical AB, Askim, Sweden) were fitted before implantation.

All patients met the following criteria for Bonebridge BCI 602 implantation: age minimum 5 years, stable bone conduction thresholds of ≤45 dB HL (pure-tone average 0.5–4 kHz), anatomical suitability for placement of the internal implant component, verified by computed tomography, realistic expectations by the patient and/or their caregivers regarding device performance.

On the day of processor activation and at the 3–6-month follow-up, patients underwent pure-tone and speech audiometry. The study protocol was and approved by the Institutional Review Board of Institute of Physiology and Pathology of Hearing (IFPS:/KB/12/2024).

### 2.3. Surgical Procedure

Bonebridge implantation surgery was performed under general anesthesia. Before surgery, a detailed analysis of the patient’s CT scan was performed to plan the placement of the internal component of the Bonebridge system. Particular attention was paid to the size of the mastoid process and the distance between the sigmoid sinus, dura mater, and the surface of the temporal bone. This analysis was essential for selecting the optimal position of the subcutaneously implanted active component. The temporal bone anatomy was correlated with the condition of the overlying skin and subcutaneous tissue. After a meticulous assessment of preoperative images, a decision was made regarding the proper placement of the device. In rare diseases, there is a high need to exclude areas of skin that are either too thin or too thick. There is also a need to analyze the vessels (e.g., emissary veins) and the thickness of the squamous part of the temporal bone. In all cases, the device’s position was placed 10 mm higher than recommended due to thin skin and subcutaneous tissue, in order to prevent potential extrusion. In those cases where the soft tissue thickness exceeded 7–8 mm and there was risk of insufficient magnet retention, thinning of the subcutaneous tissue was done. Surgical access was achieved via a posterosuperior C-shaped skin incision, followed by careful dissection of the subcutaneous tissue and periosteum.

The next step involved preparing a well for the implant within the mastoid process or above the temporal line. The implant was considered well placed within the temporal bone when the portion intended for insertion into the bone was 4.5 mm. In 8 patients who had craniofacial malformations, full placement of the implant within the temporal bone was not possible because the bone was too thin. In such cases, the implant was positioned partially (n = 4), and in some cases, completely (n = 4), in contact with the dura mater or sigmoid sinus.

Following preparation of the implant well and insertion, the active component was angled up to 120°, although exceeding 90° is not recommended by the manufacturer. The implant was secured using supplied self-tapping silver screws measuring 1.6 × 5 mm, and in 3 cases where there was excessively cancellous bone, with an extra rescue screw of 1.9 × 5 mm. Due to the risk of skin complications and elevation caused by the implant (which protrudes 4.2 mm above the temporal bone surface), washers or pads were not used. After achieving hemostasis, the surgical wound was closed in a layer of subcutaneous sutures followed by a layer of continuous skin closure (Polysorb 2-0 or Monosoft 3-0). To reduce the risk of hematoma, a pressure dressing was applied for 1–3 days postoperatively. Sutures were removed between postoperative days 7 and 14. Standard antibiotic prophylaxis with amoxicillin–clavulanate was also administered postoperatively. When the external dressings and sutures were removed, meticulous care of the incision site using moisturizing and antiseptic agents was taken. Long-term follow-up is advised for patients with dermatologic conditions, diabetes mellitus, or a history of previous surgery in the same anatomical region. These recommendations were applied to the patients from the study group.

## 3. Results

### 3.1. Demographic Data and Anatomical Assessment

The study was based on 10 patients (6 women, 4 men) aged 6–45 years, all diagnosed with congenital syndromes affecting the external and/or middle ear. In one case (patient 10), in addition to middle ear malformation, inner ear abnormalities were also present. This patient had mixed hearing loss and met the criteria for Bonebridge implantation. Therefore, this option was considered appropriate and potentially beneficial. Details are presented in Table 1.

### 3.2. Bonebridge Implantation Procedure

**Figure 1:**
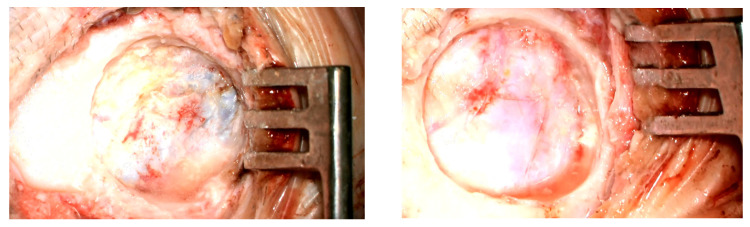
Bed in temporal bone with partial contact with dura mater.

Implantation was successfully performed in all patients. The most common intraoperative finding was contact between the implant and the dura mater. Intraoperative photos of patients of whom the bone bed prepared for the implant was in contact with dura mater are presented in Figure 1, and this was observed in 8 patients. The mean contact area, calculated for the entire study group (including patients without confirmed contact), was 70% of the implant surface. Compression of the sigmoid sinus was observed in 2 patients. No major intraoperative complications were recorded; however, heavy bleeding from the subcutaneous tissues occurred in 2 cases. Detailed surgical findings are presented in Table 2.

### 3.3. Preoperative Audiological Assessment

Prior to Bonebridge implantation, all patients were diagnosed with conductive or mixed hearing loss. The most common type was conductive hearing loss associated with ossicular malformations and/or external auditory canal atresia. The mean air-conduction threshold (PTA AC, 0.5–4 kHz) ranged from 48.75 to 90 dB HL, while the mean bone-conduction threshold (PTA BC) ranged from 10 to 36.25 dB HL. Detailed audiological results are presented in Table 3.

### 3.4. Postoperative Audiological Assessment

**Figure 2:**
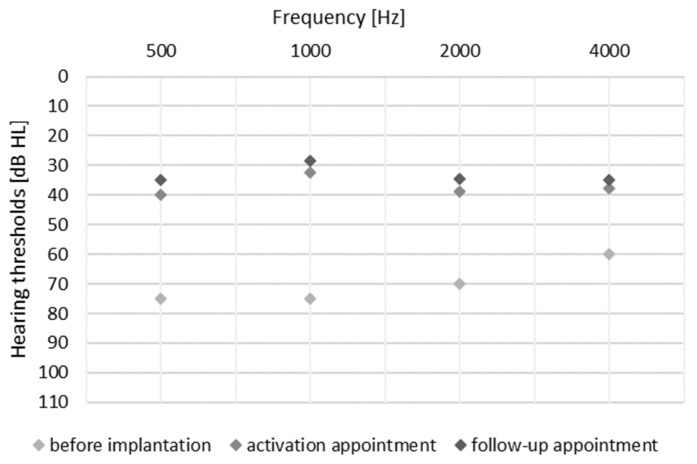
Mean free-field hearing thresholds with contralateral masking measured at three time points: before implantation, on the day of sound processor activation, and at the 3–6-month follow-up.

The effectiveness of the Bonebridge implant was assessed under free-field conditions using an active sound processor with contralateral ear masking. This was done prior to surgery, on the day of device activation, and at follow-up 3–6 months post-activation. Audiological evaluation included measurement of hearing thresholds and speech recognition testing at 65 dB SPL. All patients demonstrated improved PTA4 free-field hearing thresholds from unaided M = 68.25 (SD = 12.5) to 37.5 (SD = 4.25) on the day of processor activation, and with a slight additional improvement at the follow-up M = 33.8 (SD = 3.8). Hearing thresholds for each frequency is shown in Figure 2. Mean speech recognition scores increased from 2% (SD = 5.77) preoperatively to 78.63% (SD = 9.02) on the day of activation and further to 86.25% (SD = 13.39) at follow-up.

## 4. Discussion

The Bonebridge bone conduction implant has been shown to be an effective and safe treatment option for conductive and mixed hearing loss in patients with congenital malformations of the external and middle ear, such as microtia and external auditory canal atresia. These deformities may occur as isolated anomalies or as part of genetic syndromes, including TCS, CHARGE syndrome, KFS, and GS. Conventional air-conduction hearing aids are often ineffective or impossible to use in such cases, whereas the Bonebridge system can allow effective auditory rehabilitation. Surgical implantation of bone-conduction devices in patients with rare genetic syndromes is technically challenging due to their complex craniofacial anatomy and limited space available for implantation. The present results align with the existing literature, and confirm the Bonebridge system’s safety and audiological efficacy in this patient group. In the presented cases, bone conduction implants were selected as the safest and most effective treatment option. Alternative approaches, such as middle ear reconstruction or conventional middle ear implants, were considered less suitable due to anatomical limitations and greater surgical complexity. In particular, the use of middle ear implants represents a more surgically demanding option and was not considered optimal for these patients. Additionally, conventional air conduction hearing aids could not be used due to the anatomical conditions of the external auditory canal and middle ear. These devices may also cause an occlusion effect and, at higher levels of amplification, sound distortion, which further limits their effectiveness. External bone conduction devices (e.g., worn on a headband) were also considered; however, they are associated with poorer sound quality and reduced comfort compared to implantable solutions. Importantly, in patients with an air–bone gap exceeding 30 dB HL, bone conduction implants are widely recommended as an effective treatment option. In all cases, there was a high risk of reduced effectiveness of the Vibrant Soundbridge solution. Important factors included the position of the facial nerve and the size of the middle ear cavity. With earlier versions of the VSB, there was greater flexibility in positioning the FMT; however, with the current use of couplers, there is a higher risk of difficulty in adapting the device to the cavity. Additionally, the size of the round window was sufficient to allow direct placement on the round window. Therefore, taking into account both anatomical and audiological factors, bone conduction implants represented the most appropriate therapeutic choice for this group of patients.

The literature indicates that patients with TCS frequently present with significant auricular deformities, external auditory canal atresia, and middle ear hypoplasia, which often precludes the use of conventional air-conduction hearing aids or middle ear reconstructive surgery. One example is the case report of two children with TCS, a 7-year-old boy and a 6-year-old girl, who were successfully implanted with Bonebridge bone-conduction devices 17. Both patients were diagnosed with conductive hearing loss and had been previously fitted with bone-conduction hearing aids on a headband, which demonstrated positive outcomes under clinical testing. However, continuous daily use of the headband was uncomfortable and socially restrictive, prompting the decision to pursue an implantable solution. After Bonebridge implantation, both patients experienced substantial improvement in hearing thresholds and ease of use, without clinically significant intraoperative or postoperative complications. The mean unaided pure-tone average (PTA4, 0.5–4 kHz) was 65 ± 3.54 dB HL. Use of bone-conduction hearing aids on a headband improved the threshold to 30.5 ± 2.94 dB HL, whereas after implantation the mean threshold improved further to 25 ± 4.27 dB HL. Similarly, the speech recognition threshold improved markedly, from 64 ± 2 dB SPL unaided to 25 ± 5 dB SPL with the Bonebridge implant [20]. These results are consistent with those reported here. Similar benefits have been documented in other publications, confirming the effectiveness of Bonebridge implantation for patients with malformations of the external and middle ear. This includes both pediatric and adult patients with genetic syndromes. In such cases, improvements in hearing thresholds typically range from 25 to 35 dB HL. Following Bonebridge implantation, patients show substantial enhancement in free-field hearing and increased comfort with daily auditory functions without experiencing any clinically significant surgical complications [33,34,35].

Similar findings have been reported in patients with CHARGE syndrome, who frequently present with structural abnormalities of the skull, sinuses, and external auditory canal, as well as conductive or mixed hearing loss. Previous studies indicate that careful preoperative planning and precise Bonebridge implantation can markedly improve patients’ quality of life, positively influencing social and educational functioning, particularly in children and adolescents, even in the presence of complex anatomical anomalies [36].

The presented cases demonstrate outcomes similar to those reported previously, where there have been significant improvements in hearing thresholds and speech recognition. As measured by the validated subjective assessment tools (SSQ, APHAB) used in some studies, there appear to be high levels of satisfaction among patients and their caregivers. In patients with challenging anatomical conditions, the use of three-dimensional imaging and preoperative planning systems (such as Otoplan) enables good implant positioning. This minimises the risk of adverse events and enhances device effectiveness [32,37].

The Bonebridge system is a fully subcutaneous implant that, compared to older transcutaneous systems, provides an aesthetic advantage and reduces the risk of soft-tissue complications. This benefit is particularly relevant for patients with genetic syndromes who often have an increased susceptibility to infection and impaired tissue healing. In terms of long-term use and surgical outcomes, the Bonebridge system has advantages over transcutaneous implants such as the BAHA Connect. Among patients with mixed hearing loss, discontinuation of the BAHA Connect implant has been associated with postoperative complications such as skin granulation, infection, and wound-healing difficulties. In one comparison, 90% of Bonebridge recipients reported continued use compared to 71% of BAHA Connect users [38].

The literature reports numerous cases of patients with microtia and/or external auditory canal atresia who achieved substantial audiological improvements following Bonebridge implantation. One Taiwanese study included 41 patients with microtia and external auditory canal atresia (26 bilateral, 15 unilateral) who received a Bonebridge BCI 601 implant 57. The mean age at implantation was 18.9 years, and the average follow-up period was 6.3 years. Significant improvements were observed in both objective and subjective hearing parameters. Functional hearing gain averaged 34.2 ± 11.7 dB HL, and the mean free-field hearing threshold improved from 65.3 ± 8.8 dB HL without the device to 31.1 ± 9.1 dB HL with the Bonebridge implant. Speech recognition thresholds in quiet improved from 58.3 ± 7.4 dB HL to 29.4 ± 7.0 dB HL, and in noise from −1.4 ± 7.3 dB SNR to −9.6 ± 5.4 dB SNR. Word recognition scores increased from 46.4 ± 26.9% to 93.8 ± 3.1% in quiet and from 46.7 ± 21.8% to 72.7 ± 19.3% in noise. Subjective evaluation using four validated questionnaires (APHAB, SSQ, IOI-HA, and SADL) confirmed clinically meaningful improvements in hearing quality and patient satisfaction [33].

Further studies have confirmed the efficacy of Bonebridge implantation in patients with congenital craniofacial anomalies involving the external and middle ear [39,40,41].

Compared with the results obtained in patients without diagnosed genetic syndromes in the study by Sprinzl et al. (2023) [29], our group showed higher (worse) baseline hearing thresholds (68.3 vs. 55.4 dB HL). Hearing thresholds with the device during follow-up also remained slightly worse (33.8 vs. 29.9 dB HL). At the same time, a very significant improvement in speech understanding was observed—from 2% to 86.3%—whereas in the study by Sprinzl et al. [29], this increase ranged from 8.5% to 76.5%. These results indicate that appropriate qualification and use of the Bonebridge system provide significant benefits also in patients with genetic syndromes and additional comorbidities. Despite poorer sound detection thresholds, these patients can achieve very good speech recognition outcomes.

Future studies should be designed with a more detailed focus on surgical aspects, as this appears essential for understanding the factors influencing both the choice of surgical technique and patient selection. This is particularly relevant in anatomically complex cases, where the surgeon’s experience plays a key role in achieving optimal outcomes.

## 5. Conclusions

Based on the 10 case studies reported here, the Bonebridge implant appears to be an effective and safe option for auditory rehabilitation in patients with hearing loss resulting from rare genetic syndromes involving craniofacial defects. However, this challenging patient population requires individual assessment, interdisciplinary evaluation, and careful surgical planning.

## Figures and Tables

**Table 1:** Demographic data and assessment of the external and middle ear.

Patient	Gender	Age (Years)	Diagnosis	Microtia	Atresia	Middle Ear
1	F	19	TCS	Right-sided	Bilateral	Bilateral ossicular chain malformations
2	M	45	TCS	Absent	Absent	Abnormal tympanic membrane anatomy with disturbed ossicular proportions
3	F	6	TCS	Bilateral	Left-sided	Bilateral ossicular chain malformations
4	M	9	TCS	Right-sided	Bilateral	Bilateral ossicular chain malformations
5	F	7	TCS	Bilateral	Bilateral	Severely reduced middle ear space; bilateral severe hypoplasia of the incus and malleus
6	F	12	GS	Right-sided	Right-sided	Right-sided deformity of the Eustachian tube
7	M	44	GS	Left-sided	Left-sided	Hypoplasia of the tympanic cavity combined with the absence of middle ear ossicles
8	F	12	KFS	Right-sided	Absent	Right-sided deformed tympanic cavity with altered ossicular topography
9	M	12	MFDM	Bilateral	Stenosis of the right external ear canal	Deformed tympanic cavity (reduced epitympanum, enlarged mesotympanum and hypotympanum), hypoplastic oval window, normal round window, hypoplasia of the incudal crura, elongated and narrowed stapes
10	F	15	CHARGE	Middle and inner ear malformations: bilateral inner ear hypoplasia (more pronounced on the left), suspected cochlear nerve aplasia, vestibular hypoplasia, absence of semicircular canals		

**Table 2:** Surgical evaluation of patients.

Patient	Diagnosis	Implanted Ear	Implant Contact with Dura Mater	Implant Contact/Compression on Sigmoid Sinus	Intraoperative Adverse Events	Revision
1	TCS	R	¾	compressed	heavy bleeding	-
2	TCS	R	-	-	heavy bleeding	-
3	TCS	L	complete	-	-	-
4	TCS	L	¾	-	-	-
5	TCS	R	¾	contact	-	-
6	GS	R	complete	-	-	Implant protrusion with palpable subcutaneous crepitus at the implant site
7	GS	R	complete	compressed	-	-
8	KFS	R	-	-	-	Skin thinning at the implant site with local wound inflammation
9	MFDM	R	complete	compressed	-	-
10	CHARGE	L	¾	-	-	-

**Table 3:** Preoperative audiological results.

Patient	Diagnosis	Hearing Loss Type	PTA [dB HL] (0.5–4 kHz) AC	PTA [dB HL] (0.5–4 kHz) BC
1	TCS	Conductive	66.25	22.5
2	TCS	Mixed	86.25	36.25
3	TCS	Conductive	73.75	18.75
4	TCS	Conductive	57.5	10
5	TCS	Conductive	65	22.5
6	GS	Conductive	67.5	13.75
7	GS	Mixed	90	32.5
8	KFS	Conductive	60	20
9	MFDM	Mixed	67.5	31.25
10	CHARGE	Mixed	48.75	32.5

## Data Availability

Dataset available on request from the authors.

## Abbreviations

The following abbreviations are used in this manuscript:

BCDs	Bone conduction devices
NORD	National Organization for Rare Disorders
BAHA	Bone Anchored Hearing Aid
BCI	Bone Conduction Implant
CT	Computed tomography
MRI	Magnetic resonance imaging
TCS	Treacher Collins syndrome
GS	Goldenhar syndrome
KFS	Klippel-Feil syndrome
MFDM	Mandibulofacial dysostosis
AC	Air conduction
BC	Bone conduction
PTA	Pure tone average
APHAB	Abbreviated Profile of Hearing Aid Benefit
SSQ	Speech, Spatial and Qualities of Hearing
IOI-HA	International Outcome Inventory for Hearing Aids
SADL	Satisfaction with Amplification in Daily Living
